# Descriptive and predictive analysis identify centenarians' characteristics from the Basque population

**DOI:** 10.3389/fpubh.2022.1096837

**Published:** 2023-01-25

**Authors:** Sara Cruces-Salguero, Igor Larrañaga, Javier Mar, Ander Matheu

**Affiliations:** ^1^Cellular Oncology Group, Biodonostia Health Research Institute, San Sebastian, Spain; ^2^Osakidetza Basque Health Service, Debagoiena Integrated Healthcare Organisation, Research Unit, Arrasate-Mondragón, Guipúzcoa, Spain; ^3^Kronikgune Institute for Health Services Research, Barakaldo, Spain; ^4^Epidemiology and Public Health Department, Biodonostia Health Research Institute, Donostia-San Sebastián, Guipúzcoa, Spain; ^5^IKERBASQUE, Basque Foundation for Science, Bilbao, Spain; ^6^Centro de Investigación Biomédica en Red de Fragilidad y Envejecimiento (CIBERfes), Carlos III Institute, Madrid, Spain

**Keywords:** centenarians, description, predictive modeling, functional, biological, medical

## Abstract

**Background:**

Centenarians exhibit extreme longevity and have been postulated, by some researchers, as a model for healthy aging. The identification of the characteristics of centenarians might be useful to understand the process of human aging.

**Methods:**

In this retrospective study, we took advantage of demographic, clinical, biological, and functional data of deceased individuals between 2014 and 2020 in Guipúzcoa (Basque Country, Spain) taken from the Basque Health Service electronic health records data lake. Fifty characteristics derived from demographic, clinical, pharmaceutical, biological, and functional data were studied in the descriptive analysis and compared through differences in means tests. Twenty-seven of them were used to build machine learning models in the predictive analysis and their relevance for classifying centenarians was assessed.

**Results:**

Most centenarians were women and lived in nursing homes. Importantly, they developed fewer diseases, took fewer drugs, and required fewer medical attendances. They also showed better biological profiles, exhibiting lower levels of glucose, hemoglobin, glycosylated hemoglobin, and triglycerides in blood analysis compared with non-centenarians. In addition, machine learning analyses revealed the main characteristics of the profiles associated with centenarians' status as being women, having fewer consultations, having fewer diagnoses of neoplasms, and having lower levels of hemoglobin.

**Conclusions:**

Our results revealed the main characteristics linked to centenarians in the Basque Country using Computational Biology programs. These results expand the knowledge on the characterization of the centenarian population and hence of human longevity.

## Introduction

Population aging represents a first-magnitude socioeconomic challenge since the aged population is constantly increasing all over the world. Aging is a systemic, multifactorial, and degenerative process characterized by the decline and loss of physical and mental capacities (1, 2). It is accompanied by severe comorbidities, diseases, and dependence, frequent in the last life stages, which are important causes of disability and chronicity. However, different individuals display different aging trajectories.

Even though it is a controversial topic (3–5), there is evidence postulating that the maximum age of human life is located around 115–120 years (3), and more people are reaching these ages nowadays, with the number of centenarians growing rapidly, especially in advanced countries (6).

Centenarians are a population group that exhibits extreme longevity. There are two main hypotheses to explain the long life of centenarians. The first one is the “compression of morbidity” theory, which suggests that morbidity and disability would be compressed toward the end of life (7). In this line, epidemiological and longitudinal studies in cohorts such as the Long Life Family Study (LLFS) (8), the New England Centenarian Study (NECS) (9), the Longevity Genes Project (LGP) (10), and the Healthy Aging and Biomarkers Cohort Study (HABCS) (11), among others, have shown that centenarians exhibit medical histories with low incidence rates of age-associated common pathologies, such as vascular diseases, diabetes, Parkinson's disease, and cancer (12, 13). Moreover, some centenarians delay their first disease experience, frequently associated with mortality, until over the age of 90 years, or even they completely escape from these morbidities (10, 12, 13). In addition, centenarians have been associated with better cognitive functions and need less help with the activities of daily living in comparison with the elderly, in younger age groups (10, 12–14). Consistent with this idea, they seem to experiment with a faster decline in their functional status at the end of their lives (15). Because of this, some studies propose centenarians as a model of healthy aging (16, 17). On the other hand, the hypothesis of the “expansion of morbidity” (18, 19) suggests that additional years of life are accompanied by increased rates of disease, morbidity, and an increasing number of years spent in poor health in long-lived individuals. In this line, centenarians have been linked with a high disease burden (20) and diminished physical and cognitive functioning (21–25).

The Basque Country population is presenting a notable increase in life expectancy over the last few years. This has been associated with significant multimorbidity and its prevalence rises with age (26), which could coincide with the previously described “expansion of morbidity” of centenarians' profiles. However, the centenarian population of the Basque Country has not been analyzed in detail yet. In this study, we aimed at unraveling the main characteristics of the Basque centenarian population. For that, we explored a population of 47,656 individuals deceased in Guipúzcoa (Spain) between 2014 and 2020, which included records of 379 centenarians.

## Materials and methods

### Study population and design

Basque country contains 2.3 million individuals (Supplementary Figure 1) and Osakidetza is a public-funded regional healthcare system, which follows a funding model Beveridge style as English National Health Service. This study was based on a population of deceased individuals between 1 January 2014 and 31 December 2020 recorded in the data lake of the Basque Health Service, which contains information from 2004 onward. All the information registered in the database was anonymized, and it included demographic, clinical, and functional data. Before performing the analyses, the quality of the data was evaluated to delete patients whose data did not fulfill the quality criteria. Patients whose date of birth and/or death did not have a valid format (dd/mm/yyyy) or had missing values were excluded and not taken into account, as well as patients whose date of birth was posterior to the date of death.

After this data cleansing, a total of 47,656 individuals remained. For each individual, information about sex, age, province, status in nursing home, diagnoses, use of resources, pharmaceutic prescriptions, laboratory analyses, and functional measurements was included. Age was calculated as the difference between the date of death and the date of birth. For this study, we divided the population into two main groups: those individuals who were younger than 100 years at the time of decease (47,277 individuals, 99.2%) were considered non-centenarians and those who were 100 years or older (379 individuals, 0.8%) were considered centenarians (Supplementary Figure 2).

The database included information about the diagnoses performed in all the episodes of primary care, emergency, outpatient, and in-hospital care and was recorded through the International Classification of Diseases, Ninth Revision (ICD-9) and Tenth Revision (ICD-10) diagnosis codes. Regular expressions were used to sort diagnoses into ICD-9 and ICD-10 main 17 categories. The use of resources was analyzed by counting each attendance to Primary Care Nurse, Primary Care Physician, Outpatients, Home Hospitalization, Hospitalization, Procedures, Intensive Care Unit (ICU), and Emergencies. Procedures were also classified according to the ICD-9 code through regular expressions. Drugs were classified according to the Anatomical Therapeutic Chemical (ATC) code with the same method, and the total number of prescribed drugs for each individual was also calculated.

The results of the last blood analysis were included when available and had these parameters: high-density lipoproteins (HDL), low-density lipoproteins (LDL), total cholesterol, creatinine, glucose, hemoglobin, glycosylated hemoglobin, International Normalized Ratio (INR), platelets, potassium, sodium, triglycerides, and urea. Outliers were filtered when it proceeded, based on the normal ranges of values for each variable. Many patients had no analysis recorded or these were incomplete.

Levels of independence were recorded in some patients through the Barthel Index, a measurement to assess performance in activities of daily living (27). These measurements were presented in the original database through Barthel points or Barthel scale, depending on the patient. Barthel points could go from 0 to 100; the closer to 100, the greater the independence. Barthel's scale had five stages, defined by the previous scoring. To unify the information, Barthel's points were grouped into five stages of the Barthel scale. The suffered falls were also registered if any, and this was transformed into a binary variable for each individual.

### Statistical analysis

*Descriptive statistics* were applied to analyze the demographic, clinical, and functional information of centenarians and non-centenarians. For comparisons between the two groups, Student's *t*-test and Pearson's chi-square test were used for continuous and categorical variables, respectively. A two-sided *p*-value of <0.05 was considered statistically significant.

*Predictive classification models* of being centenarians were built with the variables that surpassed the previous threshold. Due to the high imbalance of classes (379:47,277), cross-validation-based and bootstrapping-based approaches were applied, and all the models were built with random subsamples from non-centenarian and centenarian populations. Training datasets were built with 450 NC and 300 C, with the possibility of duplicating individuals, and test datasets were built with 150 NC and 100 C, with the possibility of duplicating individuals. This random resampling was repeated through 100 iterations to ensure the generalization of the models. A seed was set for initializing the pseudorandom number generator to ensure the repeatability of the analyses.

First, missing values in the database were calculated through iterative imputation (Bayesian ridge estimator). Because of the potential collinearity between some of the variables, Lasso regression and Recursive Feature Elimination (RFE) with cross-validation were applied. Variables that were eliminated in >50% of resampling iterations were removed. The remaining variables were used for building logistic regression and support vector machine (SVM) models. Odds ratio (OR) with a 95% confidence interval (CI) was set as an association measure for logistic regression, and permutation feature importance was calculated for SVM. Balanced accuracy, sensitivity, specificity, precision, area under the curve (AUC), no information rate, and Cohen's kappa coefficient (κ) were calculated to test the performance of the two models in training and validation datasets, and the models with the best scores were selected. All data analyses were performed in Python 3.9, through *pandas, SciPy*, and *scikit-learn* libraries.

### Ethics

This study was approved by the Basque Clinical Research Ethics Committee (CEIm-E code PI2020206) and adhered to the tenets of the Declaration of Helsinki by the World Medical Association regarding human experimentation.

## Results

### Demographic data of centenarians

The population included in this study was formed by the 47,656 individuals deceased between 2014 and 2020 accounted in Osakidetza Basque electronic health system, 379 (0.8%) being centenarians. Among the rest of the 47,277 individuals (99.2%), 12,309 were between 90 and 99 years (25.83%), 18,701 between 80 and 89 years (39.24%), 8,794 between 70 and 79 years (18.45%), 4,999 between 60 and 69 years (10.49%), 2,752 between 50 and 59 years (5.77%), and 82 were younger than 50 years (0.17%) at the time of decease. The oldest individual was 109 years old, while the youngest one was 49.

A comparison of the demographic characteristics between centenarians and non-centenarians was gathered and revealed that most centenarians were women (85.75%), showing a significant difference compared with the group of non-centenarians in which the division of sexes was far more balanced (*p* < 0.001) (Table 1). Almost all the population from the study was concentrated in Guipúzcoa (*n* = 46,336), followed by the rest of the Basque Country (*n* = 1,224) and other provinces (*n* = 15). There were 81 individuals whose province was not specified. The distribution of both groups across the provinces did not show any significant difference (*p* = 0.99). Moreover, a significantly higher proportion of individuals who were in the nursing home was present in the group of centenarians (*p* < 0.001), while the proportion of cases that had a caregiver was also higher in this group (*p* = 0.054) (Table 1).

**Table 1:** Demographic features of centenarians and non-centenarians. Number of individuals and percentages (%).

	**NC (*n* = 47,277)**	**C (*n* = 379)**	***p*-value**
**Demographic data**			
Sex	23,959 women (50.68%) 23,318 men (49.32%)	325 women (85.75%) 54 men (14.25%)	**<0.001**
Province	45,967 Guipúzcoa (97.23%) 1,216 Rest of Basque Country (2.57%) 15 Others (0.03%)	369 Guipúzcoa (97.36%) 8 Rest of Basque Country (2.11%) 0 Others (0%)	0.999
Nursing home	41,138 no (87.01%) 6,139 yes (12.99%)	282 no (74.41%) 97 yes (25.59%)	**<0.001**
Caregiver	22,438 yes (47.46%) 2,930 no (6.2%)	197 yes (51.98%) 15 no (3.96%)	0.054

NC, non centenarians; C, centenarians.

### Clinical features of centenarians

To characterize centenarians focusing on clinical diagnoses, we reunited the mean total diagnoses for each International Classification of Diseases Ninth Revision (ICD-9) category, for all individuals during their medical records and compared centenarians and non-centenarians. Notably, non-centenarians presented more diagnoses in every single ICD-9 category, as well as in all of them (*p* < 0.001), when compared with centenarians (Table 2). Among the single ICD-9, the difference between both groups was significant in the case of neoplasms, diseases of the nervous system and sense organs, diseases of the circulatory system, and diseases of the digestive system, all of them well-known to appear in the aged population and increase with age, whereas there were no significant differences between the two groups for Infection and parasitic diseases (*p* = 0.138), injury and poisonings (*p* = 0.11), congenital anomalies (*p* = 0.319), complications of pregnancy, childbirth, and the puerperium (*p* = 0.5798), and certain conditions originating in the perinatal period (*p* = 0.84). Although the elderly (>65 years) are more prone to parasitic infections along with children (28), the impact of aging in these types of diseases is very low. In addition to those diagnoses, ICD-9 includes the category of Supplementary Factors Influencing Health Status and Contact with Health Services, which were also markedly reduced in the centenarian population (Table 2).

**Table 2:** Total diagnoses for centenarians and non-centenarians. Mean ± SD.

**Diagnoses**	**NC**	**C**	***p*-value**
Infection and parasitic diseases	0.027 ± 0.28	0.005 ± 0.07	0.138
Neoplasms	1.35 ± 4	0.08 ± 0.42	**<0.001**
Endocrine, nutritional and metabolic diseases, and immunity disorders	0.85 ± 3.78	0.25 ± 1.28	**0.002**
Diseases of blood and blood forming organs	0.2 ± 1.15	0.069 ± 0.44	**0.025**
Mental disorders	0.29 ± 1.52	0.1 ± 0.45	**0.017**
Diseases of the nervous system and sense organs	0.56 ± 1.96	0.23 ± 0.95	**<0.001**
Diseases of the circulatory system	2.06 ± 7.54	0.69 ± 2.58	**<0.001**
Diseases of the respiratory system (including Covid in 2020)	0.67 ± 3.2	0.35 ± 1.93	**0.05**
Diseases of the digestive system	0.49 ± 2.36	0.14 ± 0.76	**<0.001**
Diseases of the genitourinary system	0.52 ± 2.36	0.21 ± 1.03	**0.01**
Complications of pregnancy, childbirth, and the puerperium	0.002 ± 0.08	0 ± 0	0.58
Diseases of the skin and subcutaneous tissue	0.12 ± 0.8	0.02 ± 0.18	**0.026**
Diseases of the musculoskeletal system and connective tissue	0.31 ± 1.63	0.11 ± 0.64	**0.015**
Congenital anomalies	0.01 ± 0.21	0 ± 0	0.319
Certain conditions originating in the perinatal period	0.0001 ± 0.1	0 ± 0	0.84
Symptoms, signs, and Ill-defined conditions	0.43 ± 1.58	0.19 ± 0.97	**0.002**
Injury and poisoning	0.35 ± 1.28	0.25 ± 0.92	0.11
Supplementary factors influencing health status and contact with health services	2.41 ± 8.16	0.86 ± 2.95	**<0.001**

NC, non centenarians; C, centenarians.

We next compared the recorded use of resources, which included hospitalizations, outpatients, primary care physician visits, primary care nurse visits, emergencies, and ICU among centenarians and non-centenarians. Centenarians showed less use of all resources except for the primary care nurse, though this showed a tendency but was not enough to be significantly different (*p* = 0.12). Among them, greater differences were observed in the use of outpatients, hospitalizations, and emergencies (all of them, *p* < 0.001) (Table 3). It is also remarkable that there was no record of centenarians who entered the ICU in this study. The procedures including different types of surgical operations and diagnostic/therapeutic procedures showed the same trend, with centenarians having far fewer surgical operations and diagnostic/therapeutic procedures than non-centenarians. Indeed, among the 15 recorded procedures, only operations on the musculoskeletal system (*p* = 0.229) and obstetrical procedures (*p* = 0.9155) did not show significant differences (Table 3).

**Table 3:** Use of resources for centenarians and non-centenarians. Mean ± SD.

**Use of resources**	**NC**	**C**	***p*-value**
Primary care nurse	76.5 ± 83.8	83.24 ± 97.89	0.12
Primary care physician	105.77 ± 74.67	91.72 ± 76.51	**<0.001**
Outpatients	31.52 ± 34.35	7.2 ± 11.02	**<0.001**
Hospitalizations	33.36 ± 43.65	12.38 ± 17.17	**<0.001**
Hospitalizations at home	1.84 ± 7.25	0.81 ± 3.04	**0.006**
ICU	0.09 ± 0.34	0 ± 0	**<0.001**
Days at ICU	0.58 ± 3.57	0 ± 0	**0.002**
Emergencies	4.39 ± 5.34	2.39 ± 3.51	**<0.001**
**Procedures**			
Operations on the eye	0.95 ± 2.33	0.29 ± 1.2	**<0.001**
Other miscellaneous diagnostic and therapeutic procedures	0.08 ± 0.41	0 ± 0	**<0.001**
Operations on the ear	0.01 ± 0.17	0 ± 0	0.252
Operations on the nose, mouth, and pharynx	0.01 ± 0.55	0.01 ± 0.1	**0.021**
Operations on the respiratory system	0.26 ± 1.1	0.01 ± 0.1	**<0.001**
Operations on the cardiovascular system	0.63 ± 2.11	0.08 ± 0.66	**<0.001**
Operations on the hemic and lymphatic system	0.19 ± 0.79	0.01 ± 0.1	**<0.001**
Operations on the digestive system	1.06 ± 2.79	0.15 ± 0.81	**<0.001**
Operations on the urinary system	0.3 ± 1.53	0.04 ± 0.26	**<0.001**
Operations on the male genital organs	0.06 ± 0.38	0 ± 0	**0.004**
Operations on the female genital organs	0.12 ± 0.7	0.01 ± 0.1	**0.001**
Operations on the musculoskeletal system	0.51 ± 1.55	0.41 ± 0.95	0.229
Operations on the integumentary system	0.17 ± 0.89	0.07 ± 0.49	**0.036**
Obstetrical procedures	0.01 ± 0.12	0.01 ± 0.01	0.916
Miscellaneous diagnostic and therapeutic procedures	7.09 ± 11.33	2.68 ± 5.15	**<0.001**

NC, non centenarians; C, centenarians.

To finally characterize the clinical features of the centenarians, we compared the use of prescribed drugs. In line with the aforementioned information, centenarians displayed a general tendency of taking fewer drugs showing a significant difference in the total Number of Drugs (mean = 27.36), compared with the non-centenarian individuals (mean = 37.62) (*p* < 0.001) (Table 4). The reduction was observed for every group of the ATC classification, except for the category “Various Drugs” (mean centenarians = 0.41 vs. mean non-centenarians = 0.36), even though in this case, the difference was not significant. Furthermore, significant differences were shown for 9 of 13 ATC categories, with the exceptions of dermatologicals (*p* = 0.06), antiparasitic products, insecticides, and repellents (*p* = 0.09), anti-infectives for systemic use (*p* = 0.32), and sensory organs drugs (*p* = 0.34).

**Table 4:** Prescribed drugs for centenarians and non-centenarians. Mean ± SD.

**Prescribed drugs**	**NC**	**C**	***p*-value**
Alimentary tract and metabolism drugs	4.9 ± 5.01	3.13 ± 3.39	**<0.001**
Blood and blood forming organs drugs	2.67 ± 3.27	1.82 ± 2.39	**<0.001**
Cardiovascular system drugs	5.16 ± 5.56	3.55 ± 4.02	**<0.001**
Dermatologicals	1.62 ± 3.12	1.33 ± 2.54	0.065
Genitourinary system and sex hormones drugs	0.51 ± 1.23	0.19 ± 0.73	**<0.001**
Systemic hormonal preparations, excluding sex hormones and insulins	1.55 ± 2.75	0.82 ± 1.73	**<0.001**
Antiinfectives for systemic use	4.55 ± 5.98	4.25 ± 6.12	0.322
Antineoplastic and immunomodulating drugs	0.25 ± 1.04	0.08 ± 0.43	**0.002**
Musculoskeletal system drugs	1.8 ± 2.56	0.88 ± 1.6	**<0.001**
Nervous system drugs	10.25 ± 10.25	7.63 ± 7.75	**<0.001**
Antiparasitic products, insecticides, and repellents	0.04 ± 0.26	0.02 ± 0.13	0.093
Respiratory system drugs	2.77 ± 4.52	2.18 ± 4.42	**0.012**
Sensory organs drugs	1.2 ± 2.5	1.08 ± 2.08	0.348
Various drugs	0.36 ± 0.95	0.41 ± 1.02	0.274
Total number of drugs	37.62 ± 29.76	27.36 ± 25	**<0.001**

NC, non centenarians; C, centenarians.

### Functional data of centenarians

Next, functional measurements of centenarians and non-centenarians were compared. Most centenarians exhibited lower values on the Barthel scale compared with non-centenarians (*p* < 0.001). Thus, only 20% of centenarians were independent or minimally dependent, against 35% of non-centenarians (Table 5). On the other hand, 40% of centenarians were totally or very dependent, compared with 19% of non-centenarians. However, regarding the recorded falls, most individuals in both groups did not have any falls (almost 90% in each group), and there was no significant difference between centenarians and non-centenarians (*p* = 0.95) (Table 5).

**Table 5:** Functional measurements for centenarians and non-centenarians.

	**NC (*n* = 47,277)**	**C (*n* = 379)**	***p*-value**
**Functional measurements**			
Barthel scale	6,350 Independent (13.43%) 9,661 Minimally Dependent (20.44%) 3,881 Partially Dependent (8.21%) 3,494 Very Dependent (7.39%) 5,466 Total Dependent (11.56%)	4 Independent (1.06%) 71 Minimally Dependent (18.73%) 42 Partially Dependent (11.08%) 52 Very Dependent (13.72%) 97 Total Dependent (25.59%)	**<0.001**
Falls registered	41,528 no (87.84%) 5,749 yes (12.16%)	332 no (87.6%) 47 yes (12.4%)	0.949

Number of individuals and percentages (%). Barthel scale ranges are 100 points for Independent, >60 for Minimally Dependent, 40–55 for Partially Dependent, 20–35 for Very Dependent, and <20 for Total Dependent.

NC, non centenarians; C, centenarians.

### Laboratory data of centenarians

Next, the last recorded blood analysis results for both groups were compared. Notably, glucose levels were significantly lower (*p* < 0.001) for centenarians by 12 mg/dl. Hemoglobin and glycosylated hemoglobin were also significantly lower (*p* < 0.001 and *p* = 0.009) by a mean value of 0.48 and 0.2 mg/dl, respectively. Triglycerides were also significantly lower (*p* = 0.002) for centenarians by around 11 mg/dl. Sodium levels showed significant differences (*p* = 0.04) with a mean value of 141.53 mEq/l in centenarians vs. 141.03 mEq/l in non-centenarians. On the contrary, there was no significant difference in HDL (*p* = 0.20), LDL (*p* = 0.65), and total cholesterol (*p* = 0.78) between the two groups. In addition, creatinine, INR, platelets, potassium, and urea levels did not show significant differences between the two groups (all of them, *p*>0.05) (Table 6).

**Table 6:** Laboratory analyses results for centenarians and non-centenarians. Mean ± SD.

**Laboratory analysis**	**NC**	**C**	***p*-value**
HDL (mg/dl)	51.12 ± 17.61	52.39 ± 17.7	0.208
LDL (mg/dl)	120.33 ± 35.96	111.46 ± 30.83	0.654
Total cholesterol (mg/dl)	178.07 ± 47.72	177.32 ± 41.34	0.785
Glucose (mg/dl)	110.98 ± 41.61	98.98 ± 28.95	**<0.001**
Creatinine (mg/dl)	1.15 ± 1.37	1.11 ± 0.59	0.578
Hemoglobin (g/dl)	12.73 ± 2.06	12.25 ± 1.71	**<0.001**
Glycosylated hemoglobin (%)	6.26 ± 1.14	6.01 ± 0.94	**0.01**
INR	61.26 ± 44.72	53.44 ± 48.03	0.093
Platelets (billions/l)	230.59 ± 92.83	222.48 ± 71.11	0.122
Potassium (mEq/l)	4.59 ± 6.97	4.56 ± 0.55	0.691
Sodium (mEq/l)	141.03 ± 4.73	141.53 ± 4.58	**0.041**
Triglycerides (mg/dl)	114.86 ± 64.88	103.71 ± 44.06	**0.003**
Urea (mg/dl)	56.67 ± 37.74	60.3 ± 33.11	0.12

NC, non centenarians; C, centenarians.

### Predictive model

Next, we completed predictive models using machine learning and data mining to forecast the likely future outcomes of centenarians. Before that, Lasso regression and RFE with cross-validation were performed to remove collinearity and select the most relevant variables. From the initial significant 50 variables, 28 were kept after running these two algorithms. With these, two machine learning models were built, logistic regression and SVM. These approaches were considered for their performance and practical usefulness in the analysis of different types of medical data considering the dataset dimensionality and the posterior interpretability. We first run the logistic regression model from which Table 7 shows a summary of the results including OR, CI of 95%, and *p*-value. The reference for the odds ratio is computed as male individuals that did not live in the nursing home, totally dependent, with no diseases, use of resources, or drugs, and all the laboratory values set at 0. Therefore, the odds of being centenarian in women are almost two times the odds of being centenarian in the reference (Table 7). Being in a nursing home, increases 64% of the odds of being centenarian, while each step up in the Barthel Index decreases the odds by 40%. An increase in one consultation is associated with a reduction of 95%, while each home hospitalization and appointment with the primary care physician increases the odds by 177 and 169%, respectively. Furthermore, having a diagnosis of neoplasia is also related to a reduction of 76% in the odds of reaching 100 years. Each prescription of systemic hormonal preparations, excluding sex hormones, and insulins, is also associated with a reduction in the odds of being centenarian while taking respiratory system drugs increases the odds. An increase of 1 g/dl in hemoglobin levels is related to a reduction of 24% in the odds of being centenarian (Table 7). Taking into account statistical significance, the main characteristics associated with centenarians in this predictive model are consultations, sex, living in residence, Barthel Index, hospitalizations, respiratory system drugs, and family doctor visits.

**Table 7:** Results from logistic regression model. OR, CI (95%), and *p*-values, sorted by *p*-value.

**Variables**	**OR**	**CI**	***p*-value**
Consultations	0.05	0.02–0.11	<0.001
Sex	1.91	1.5–2.43	<0.001
Nursing home	1.64	1.33–2.02	<0.001
Barthel	0.6	0.47–0.76	<0.001
Home hospitalizations	1.77	1.31–2.39	<0.001
Respiratory system drugs	1.75	1.26–2.43	<0.001
Primary care physician	1.69	1.24–2.29	<0.001
Hemoglobin	0.76	0.59–0.98	0.033
Neoplasms	0.24	0.09–0.64	0.005
Systemic hormonal preparations, excluding sex hormones and insulins	0.65	0.46–0.91	0.012
Glucose	0.72	0.52–1	0.051
Triglycerides	0.83	0.67–1.04	0.104
Hospitalizations	0.68	0.38–1.22	0.199
Cardiovascular system drugs	1.22	0.9–1.65	0.203
Nervous system drugs	0.85	0.6–1.2	0.363
Operations on the eye	1.13	0.86–1.5	0.383
Glycosylated hemoglobin	1.16	0.83–1.63	0.387
Genitourinary system and sex hormones drugs	1.13	0.84–1.53	0.411
Musculoskeletal system drugs	0.88	0.64–1.22	0.441
Alimentary tract and metabolism drugs	0.89	0.6–1.32	0.554
Blood and blood forming organs drugs	0.93	0.69–1.25	0.614
Operations on the digestive system	1.06	0.77–1.47	0.707
Diseases of the nervous system and sense organs	0.97	0.7–1.34	0.842
Miscellaneous diagnostic and therapeutic procedures	1.03	0.69–1.53	0.891
Supplementary factors influencing health status and contact with health services	1.04	0.61–1.77	0.900
Sodium	1.01	0.82–1.26	0.914
Emergencies	0.98	0.69–1.53	0.923
Diseases of the circulatory system	1.01	0.66–1.56	0.953

**Figure 1:**
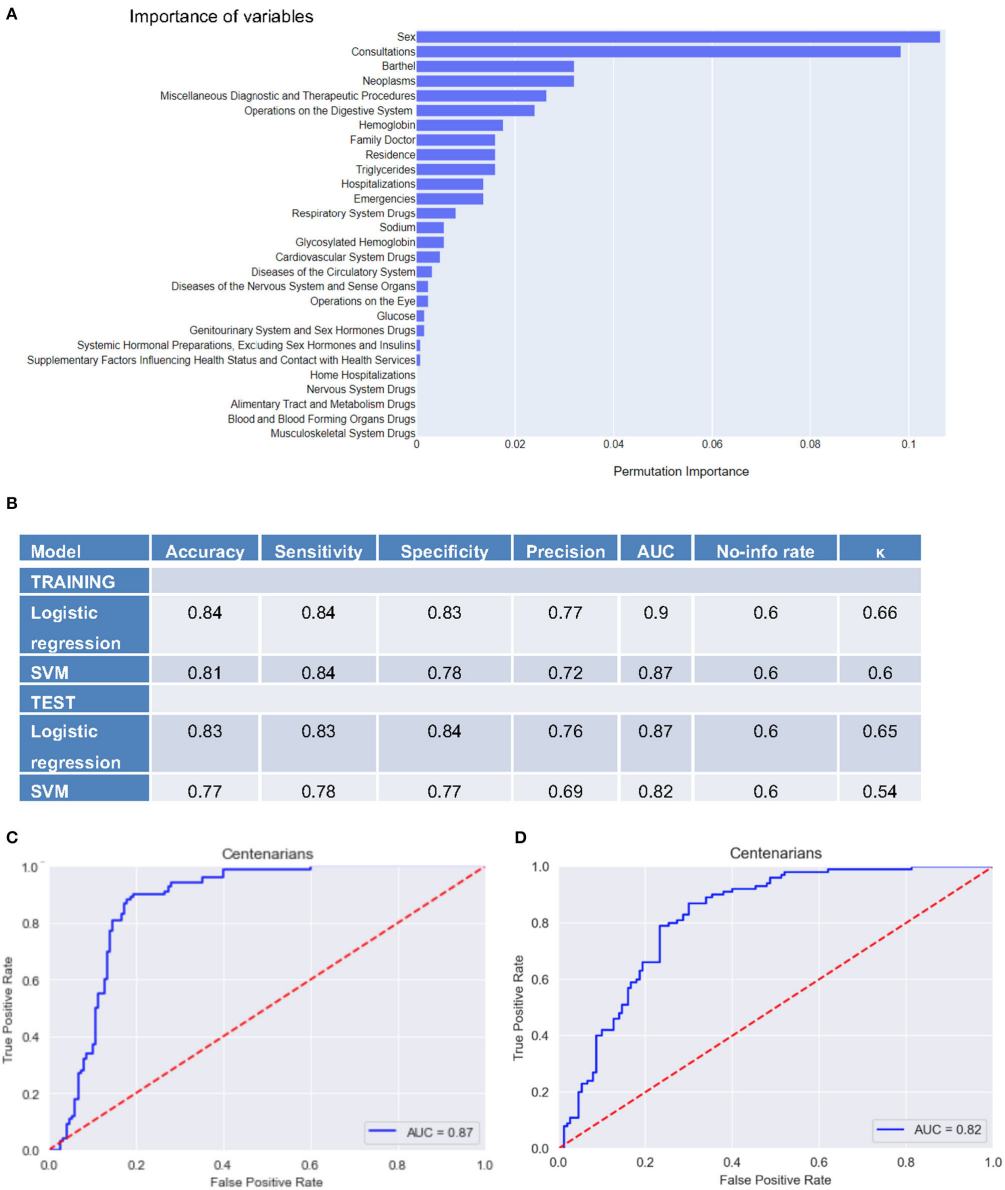
Results from machine learning models. **(A)** Permutation importance of selected variables in the SVM model. Coefficients represent the decrease in the predictions when randomly shuffling the values of a given feature. **(B)** Performance of predictive models in centenarians and non-centenarians classification in validation datasets. **(C)** Logistic regression ROC curve, with an AUC of 0.87. **(D)** SVM ROC curve, with an AUC of 0.82.

In the SVM model, the importance of the variables was assessed through permutation feature importance (Figure 1A). Sex was revealed to be the most relevant variable when classifying centenarians and non-centenarians, followed by the number of consultations, Barthel Index, and the number of neoplasms. On the other hand, the number of home hospitalizations and the drugs for the nervous system, alimentary tract and metabolism, and blood and musculoskeletal system were less relevant for the classification (Figure 1A). Next, the performance of the two models was compared and represented through various metrics, in both training and test datasets employed in machine learning (Figure 1B). The accuracy and sensitivity achieved by the models showed good discriminant capacity when classifying centenarians. Receiver Operating Characteristic (ROC) curves for both models in test datasets were built, with an AUC of 0.87 for logistic regression (Figure 1C) and 0.82 for SVM (Figure 1D). Overall, both models showed similar performance, with logistic regression being slightly better.

## Discussion

There are a few studies that analyzed the features of centenarians and relate them to human aging. Demographic, socioeconomic, cognitive, functional, and clinical data can be retrieved through the use of questionnaires (10, 12, 29), which usually focus on the assessment of vital and behavioral status or the analysis of medical expenditures (30). However, the number of studies that provide a complete picture of centenarians' profiles based on healthcare records is very scarce (31, 32). To date, there was no analysis of centenarians from the Basque Country either. In this study, we analyzed the population of 47,656 individuals deceased in Guipúzcoa between 2014 and 2020, among which there were 379 centenarians.

The descriptive analysis showed a notable difference between the sexes. Indeed, the proportion of women in the group of centenarians was far higher than in the non-centenarian group. This is consistent with the results of previous studies (11, 33), and the predictive analysis revealed that being a woman increased the odds of becoming a centenarian by nearly 100% compared with the reference group.

The presence of chronic diseases, mainly neoplasms, and cardiovascular and neurodegenerative diseases were identified as significant variables in the descriptive analysis, with centenarians displaying fewer diseases than non-centenarians. These findings are consistent with previous ones found in the analysis of New England long-lived individuals (10) and Danish (34) and Japanese centenarians (30). Interestingly, neoplasms were one of the most relevant variables in both logistic regression and SVM models, with the increase of neoplasms supposing a reduction in the odds of being centenarian. This coincides with the finding of a previous study that postulated that the hazard ratios for neoplasms and other age-related diseases were less with older age (35). The population of Basque centenarians used fewer medical resources, particularly consultations, experienced fewer hospitalizations, had fewer operations, and they also took fewer drugs. These results confirm that centenarians present better health, and this relates to their capacity of avoiding or postponing diseases until the last stages of their lives (35, 36). Thus, our results reinforce the “compression of morbidity” theory, since Basque centenarians decrease the appearance of chronic diseases, and this characteristic is one of the main characteristics of centenarians in the predictive models. Our data are also in line with the idea proposed by Evert et al., estimating that around 80% of centenarians belonged to “delayers” or “escapers” groups, with late age of onset or no history of common age-related diseases before 100 years (12). The level of independence of Basque individuals also supports the “compression of morbidity” hypothesis, with a higher proportion of centenarians being totally dependent or very dependent than non-centenarians but not higher falls. In line with this, the Barthel Index was revealed as one of the most relevant variables in both predictive models, and each level increased in the scale led to a reduction of 40% in the odds of being centenarian.

The biological characterization of blood analyses also supports the “compression of morbidity” and that centenarians live with better health. Thus, centenarians had lower glucose, hemoglobin, glycosylated hemoglobin, and triglyceride levels, and overall, the mean values of biological parameters in centenarians suggest healthier status than those of non-centenarians. In line with these results, additional studies have observed that individuals with exceptional familial longevity also showed better lipid profiles, with lower levels of triglycerides, when compared with other elderly cohorts (8), and reduced levels of blood glucose, alanine-aminotransferase (ALT), and cholesterol and platelet levels were observed in centenarians (37). The significance of the differential biological profile in centenarians was further highlighted as hemoglobin was among the most relevant variables in the SVM and logistic regression models. The results of our study, together with the above cited, indicate that there is a “rectangularization” in the morbidity curves of centenarians that seem to lead to a similar rectangularization of the survival curve itself. In the last century, population survival curves have assumed a more rectangular form due to a more pronounced increase in the average length of life than in the maximum life span (7). This change is even more notable in the case of centenarians, providing an ideal scene in which early death would be erased.

Our study has some limitations. The best study design would be to compare centenarians to individuals of the same birth cohort of centenarians but not surviving up to the age of 100 years. In this way, there would not be cohort bias. However, very few studies have compared centenarians to non-surviving members of the centenarians' birth cohort. Although no differences were detected between centenarians and non-centenarians regarding COVID-19 infection, 2020 was included when the COVID-19 pandemic started. The remarkable difference between the number of centenarians and non-centenarians produced an imbalance of classes that conditioned the posterior statistical analyses. Furthermore, the imputation of data in some records could have distorted the results of the predictive models, compared with those obtained in the first mean difference analysis. Moreover, some variables could be further explored, such as the specific diagnoses and prescriptions of each individual, or the evolution of the laboratory parameters over time. However, our results highlight centenarians' characteristics that are coherent with those already published, such as the higher prevalence of women centenarians, better levels of glucose and hemoglobin, a remarkable reduction in the number of diseases in comparison with the non-centenarian control group, and poorer levels of independence. All of these suggest that the centenarian population of Guipúzcoa spends most of their lives healthy, in comparison with the non-centenarian population supporting the “compression of morbidity” hypothesis. In summary, this study takes advantage of computational biology to further describe the centenarian population and extend the knowledge on human longevity.

## Data availability statement

The original contributions presented in the study are included in the article/Supplementary material, further inquiries can be directed to the corresponding author.

## Ethics statement

The studies involving human participants were reviewed and approved by Basque Clinical Research Ethics Committee (CEIm-E code PI2020206). Written informed consent for participation was not required for this study in accordance with the national legislation and the institutional requirements.

## Author contributions

SC-S performed analysis and wrote a draft of the manuscript. IL and JM collected clinical information from the Basque Health Service data lake, and all revised the manuscript. AM directed the project, contributed to data analysis, obtained funds, and wrote the manuscript. All authors contributed to the article and approved the submitted version.
