# Supplementary material for: Descriptive and predictive analysis identify centenarians' characteristics from the Basque population
Source: Front Public Health. 2023 Jan 25;10:1096837. doi: 10.3389/fpubh.2022.1096837 (PMC9905795; doi:10.3389/fpubh.2022.1096837)
Supplement: Supplementary file 1 [file Data_Sheet_1.pdf]

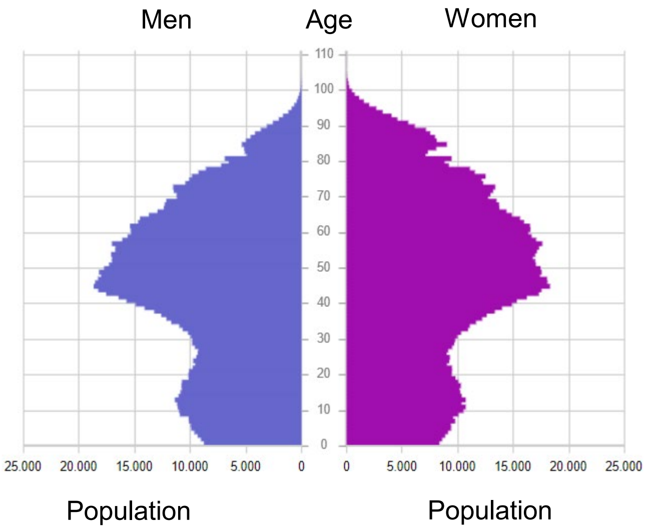

Figure Legend. 1. Basque Country population pyramid in December 2021 obtained from https://www.eustat.eus.

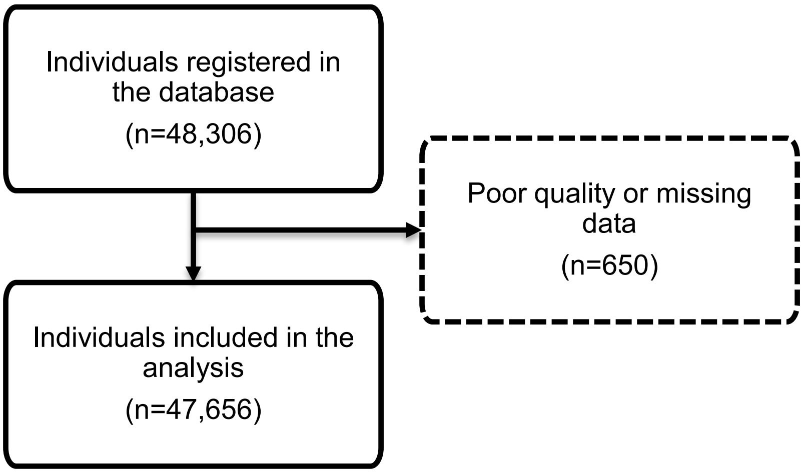

Figure Legend. 2. Flow chart with analysis of individuals studied
